# Seasonal Influences on Human Placental Transcriptomes Associated with Spontaneous Preterm Birth

**DOI:** 10.3390/cells14040303

**Published:** 2025-02-18

**Authors:** Khondoker M. Akram, Eleanor Dodd, Dilly O. C. Anumba

**Affiliations:** Division of Clinical Medicine, School of Medicine & Population Health, Faculty of Health, The University of Sheffield, Jessop Wing, Tree Root Walk, Sheffield S10 2SF, UK; k.m.akram@sheffield.ac.uk (K.M.A.); edodd2@sheffield.ac.uk (E.D.)

**Keywords:** thermal effects on placental transcriptome, climate change and preterm birth, placental inflammation in heat exposure

## Abstract

Demographic studies have revealed a strong association between exposure to high ambient temperatures during pregnancy and increased risks of preterm birth (PTB). The mechanism underlying this association is unclear, but it is plausible that altered placental function may contribute to it. In this study, we conducted differential gene expression analysis, gene set enrichment analysis (GSEA), and gene ontology (GO) analysis on bulk RNA-seq data from human placentas delivered at term and preterm during the warmer months compared to placentas delivered at term and preterm during the colder months in the UK. We detected 48 differentially expressed genes in preterm placentas delivered during the warmer months compared to preterm placentas delivered during the colder months, the majority of which were inflammatory cytokines and chemokines, including SERPINA1, IL1B, CCL3, CCL3L3, CCL4, CCL4L2, CCL20, and CXCL8. The GSEA positively enriched 17 signalling pathways, including the NF-κB, IL17, Toll-like receptor, and chemokine signalling pathways in preterm placentas delivered during warmer months. These results were not observed in the placentas delivered at term during the same times of the year. The GO analysis revealed several enhanced biological processes, including neutrophil, granulocyte, monocyte, and lymphocyte chemotaxis, as well as inflammatory and humoral immune responses in preterm placentas, but not in placentas delivered at term in the summer. We conclude that maternal exposure to warm environmental temperatures during pregnancy likely alters the placental transcriptomes towards inflammation and immune regulation, potentially leading to PTB.

## 1. Introduction

Increases in global environmental temperature owing to climate change pose significant health risks for vulnerable populations, including pregnant women with limited physiological adaptability to extreme temperatures [1,2]. The heat-associated detrimental effects likely disproportionately affect women in low- and middle-income countries (LMICs), who often continue their chores and agricultural work throughout the pregnancies [3,4]. Epidemiological studies provide strong evidence of the increased risks of pregnancy complications such as preterm birth (PTB) (<37 gestational week) from exposure to high ambient temperatures and heatwaves during pregnancy [5,6,7,8,9,10].

Globally, approximately 15 million babies are born preterm every year, and over one million of them die due to prematurity-associated complications [11]. The rate of PTB is significantly higher in women in LMICs compared to those in high-income countries (HICs). Over 81% of all preterm births occur in Asia and Sub-Saharan Africa [5,11]. More than half of all preterm births occur spontaneously without any detectable causes. Spontaneous preterm birth has strong links with exposure to extreme temperatures during pregnancy, although the underlying mechanisms remain elusive [9,12].

A moderate level of heat exposure (environmental temperature > 24.8 °C) for a shorter duration can significantly increase maternal body temperature, resulting in both maternal and foetal stress, issues potentially driven by a reduction in placental blood flow [13]. A systematic review of 70 studies from 27 countries revealed the existence of a strong link between high environmental temperatures and adverse pregnancies, including PTB. The study showed an increased risk of PTB by 5% for every 1 °C increase in temperature, with a 16% increase during heatwaves [5]. Prolonged exposure to high heat, lasting three months or more, was associated with a rise in the PTB rate of 14%, whereas exposure for less than 4 weeks was associated with a rate of 7%, suggesting a dose–response association of high temperature with PTB. A cohort study on Chinese singleton pregnancies showed the significant association of both heat and cold exposure with increased risk of PTB [9]. The 2nd and 3rd trimesters of pregnancy seem most vulnerable to ambient heat exposure, causing PTB and low birth weight [8].

The placenta plays a pivotal role in maintaining pregnancy and foetal growth. Placental dysfunction is implicated in various adverse pregnancy outcomes, including preeclampsia, foetal growth restriction, recurrent miscarriage, stillbirth, and preterm birth [14]. A study showed that maternal exposure to 29 °C during late pregnancy reduced the average size of the placenta compared to exposure to a 20 °C ambient temperature [15]. Animal model studies also showed that excessive heat exposure during pregnancy significantly reduces placental weight and diminishes placental transport [16,17]. Together, these results indicate that exposure to high temperatures during pregnancy impairs placental development and function. However, the mechanisms by which continuous heat exposure for longer periods during gestation affects placental function and contributes to PTB is unclear.

Understanding the molecular immuno-genetic changes in the placenta due to exposure to high environmental temperatures is essential for developing effective interventions, which could include biomarker prediction tests for preterm birth, targeted therapeutics, and environmental and behavioural modifications to mitigate preterm birth-associated complications, particularly in the LMICs.

To reveal the molecular changes in the placenta due to heat exposure during pregnancy, we conducted differential gene expression analyses and a comparison of RNA-seq data from human placentas (chorionic villi) delivered at term and preterm during (a) the warmer time of the year and (b) the colder time of the year. We did not identify any transcriptomic differences in the placentas delivered at term during summer warmer months compared to the placentas delivered at term during colder winter months. However, 48 genes were differentially expressed in preterm placentas delivered during warmer months compared to the preterm placentas delivered during winter months. These genes were predominantly immune and inflammatory in nature. Our gene set enrichment analysis (GSEA) revealed the positive enrichment of signalling pathways predominantly associated with inflammation, immune regulation, and graft rejection in preterm placentas delivered during the warmer months, suggesting that these inflammatory signalling pathways may play a role in preterm birth associated with heat exposure during pregnancy. To our knowledge, this is the first report showing seasonal variation in the placental transcriptome linked to preterm birth.

## 2. Materials and Methods

### 2.1. Ethical Approval

This study involving human participants was approved by the London—Fulham Research Ethics Committee, NHS Health Research Authority (18/LO/2044), UK. The patients provided their written informed consent to participate in this study. All data were anonymised using alpha-numeric codes prior to analysis.

### 2.2. Study Participants and Thermal Exposure Groups Stratification

**Figure 1:**
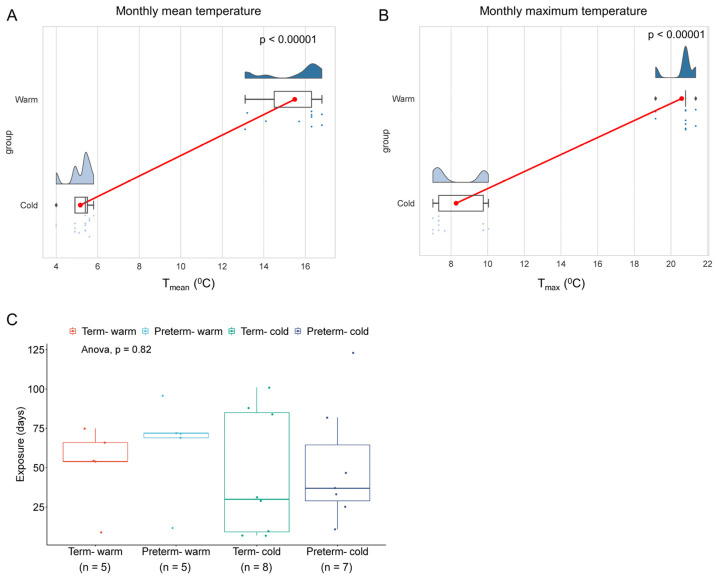
Environmental temperature exposure during pregnancy. The rain-cloud plots showing monthly mean temperature (**A**) and average maximum temperature (**B**) when the women delivered. The clouds show the data kernel density, red dots with connectors show the mean, box plots show the median with the IQR, and each dot represents an individual subject. *n* = 10 warm group and *n* = 15 cold group (**A**,**B**). *p* values were calculated by the Mann–Whitney U test. (**C**) Box blots with jitters showing the duration of exposure of 4 groups of pregnant women to warm or cold weather temperatures prior to delivery. Data are presented as median values with the IQR, and each dot represents an individual subject. *p* = 0.82, as determined by one-way ANOVA.

A total of 25 raw RNA-seq FASTQ files out of 40 RNA-seq datasets were downloaded from the NCBI Gene Expression Omnibus (GEO) repository using accession number GSE211927 based on the time of delivery (Supplementary Table S1). The datasets were divided into four groups based on the time of delivery and gestation. Women who delivered between June and September and spent at least one week in this period before spontaneous delivery at term (>37 weeks gestation) or preterm (<37 weeks gestation) were categorised as the warm-exposure group. Similarly, women who delivered between November and March and spent at least one week in this period before delivery were categorised as the cold-exposure group (Supplementary Table S1). Temperatures and exposure durations are tabulated in Table 1 and Table S1 and are presented in Figure 1. All participants resided in the South Yorkshire region in the UK during the pregnancy and immediately preceding labour. Regional weather temperatures (monthly mean and maximum temperatures) were collected from UK Met Office websites: (https://www.metoffice.gov.uk/research/climate/maps-and-data/uk-and-regional-series (accessed on 3 April 2024) and https://www.metoffice.gov.uk/research/climate/maps-and-data/uk-climate-averages/ (accessed on 3 April 2024)).

### 2.3. Differential Gene Expression (DE) Analysis

FASTQ RNA-seq files (Nanopore long reads) (NCBI SRA files) were downloaded to the Galaxy web server (Galaxy version 24.1.0.dev0) [18], and adapters were removed using the Porechop tool (v0.2.4). Reads were further trimmed with the Trim Galore tool to extract high-quality reads with a Phred score ≥ 10, which is considered to indicate good quality in Nanopore sequencing. The final read quality was assessed using pycoQC (v2.5.2). Reads that passed quality inspection were mapped onto the reference human genome (hg38) using minimap2 (v2.28) [19]. Post-mapping BAM files were used for feature counting with the featureCounts tool [20].

Next, we conducted differential gene expression (DE) analyses between warm and cold groups in term and preterm placentas using the edgeR tool (v3.36.0), with TMM data normalisation and Benjamini–Hochberg false discovery rate (FDR) correction also performed [21]. In order to identify differentially expressed genes (DEGs), an FDR (adjusted *p*-value) threshold of <0.05 and a log2FC (Fold change) cutoff of >±1 were applied. Our power analysis estimated a statistical power of 60% with *n* = 5, 74% with *n* = 7, and 80% with *n* = 8 samples, considering effect size = 2, the proportion of non-differentially expressed genes of pi0 = 0.95, and alpha = 0.05 [22,23].

### 2.4. Gene Set Enrichment Analysis (GSEA) and Gene Ontology (GO) Analysis

To explore the underlying signalling pathways, GSEA analysis was performed on the differentially expressed gene table (complete table) using the KEGG database via the WebGestalt 2024 web tool [24,25,26]. GSEA was also conducted to determine ‘cell-type’ and ‘disease phenotype’ enrichment using the Human Cell Landscape and DisGeNET databases, respectively (WebGestalt). To explore the underlying biological processes, gene ontology (GO) analysis was performed on the significantly upregulated and downregulated DEGs using the Over-Representation Analysis (ORA) enrichment method [24]. For KEGG pathway analysis between preterm-warm (*n* = 5) and term-warm placentas (*n* = 5) (Table S1), an ORA enrichment method was employed using the gProfiler web server by selecting the ‘annotated gene database’ with Benjamini–Hochberg false discovery rate mode, as described previously [19]. An FDR value of <0.05 was considered statistically significant for all enrichment analyses.

### 2.5. ELISA Assay

Protein extraction from placental villous tissue and the ELISA assay were conducted following our previously published protocol [27]. Briefly, total protein was extracted from 100 mg of snap-frozen villous tissue (biopsied from an identical location from where tissue samples were harvested for the RNA-seq analysis [19]) using a SigmaFAST/IgePal lysis buffer (Sigma-Aldrich, Cat No: I3021, St. Louis, MO, USA) with a SigmaFAST protease inhibitor cocktail (Sigma-Aldrich, Cat No: S8830) according to the manufacturer’s instructions. The tissue was homogenised using a TissueLyser LT for 10 min at 50 Hz at room temperature, followed by incubation at 4 °C for 30 min. The fully lysed tissue homogenate was centrifuged at 16,000× *g* for 20 min at 4 °C. The supernatant was collected, and the total protein concentration was determined immediately after extraction using the Qubit Protein Assay Kit (Thermo Fisher Scientific, Cat No: Q33211, Waltham, MA, USA). Samples were then diluted to a concentration of 300 μg/mL in PBS before being stored at −80 °C in a freezer in small aliquots for the ELISA assay. The quantification of the SERPINA1 protein was performed using the Human SERPINA1 ELISA Kit (ProteinTech, Cat No: KE00178, Manchester, UK) after one week of protein extraction according to the manufacturer’s instructions. A sigmoidal four-parameter logistic curve was used to generate a standard curve with which to quantify the protein concentration using GraphPad Prism (version 10.2.3).

### 2.6. Statistical Analysis

The statistical analyses and generation of plots were performed using GraphPad Prism (v10), the Galaxy web server, Cytoscape-GeneMANIA [28], and the SRplot web server [29]. The Mann–Whitney U test, Wilcoxon signed-rank test, and Student’s *t*-test were utilised to compare two groups, while one-way ANOVA was employed for comparisons involving more than two groups. A *p*-value < 0.05 was considered statistically significant. Data are presented as mean ± standard deviation (SD) or as median and interquartile ranges (IQR), as specified in the respective figure legends.

## 3. Results

### 3.1. Study Subjects and Seasonal Temperature Exposures

To investigate how summer warm environmental temperatures influence placental molecular functions, we analysed bulk RNA-seq data from our previously published research on human placentas (chorionic villi) from spontaneous term and preterm births [19]. A total of 25 raw RNA-seq FASTQ files out of 40 sequence datasets were downloaded from the NCBI Gene Expression Omnibus depository using GEO accession number GSE211927 (Supplement Table S1). Datasets were divided into four groups: (i) preterm placentas delivered during the summertime in the South Yorkshire region of the UK with a monthly mean weather temperature of 14.92 °C (T_mean_ = 14.92 °C) and a mean maximum temperature of 20.47 °C (T_max_ = 20.47 °C) (*n* = 5) (the Preterm-warm group); (ii) preterm placentas delivered during the wintertime with a T_mean_ = 5.14 °C and a T_max_ = 8.37 °C (*n* = 7) (Preterm-cold group); (iii) term placentas delivered during the summertime with a T_mean_ = 16.06 °C and a T_max_ = 20.69 °C (*n* = 5) (Term-warm group); and (iv) term placentas delivered during the wintertime with a T_mean_ = 5.18 °C and a T_max_ = 8.20 °C (*n* = 8) (Term-cold group) (Table 1 and Table S1, Figure 1). The pregnant women were exposed to warm and cold temperatures for an average of 8.3 and 6.8 weeks, respectively, preceding delivery (Table 1, Figure 1C). 

We compared gene expression and signalling pathways between Preterm-warm vs. Preterm-cold, and Term-warm vs. Term-cold placentas to evaluate the molecular changes due to warm environmental temperature exposure on placentas in preterm and term births, respectively. This strategy eliminated the potential gestational age (GA)-associated confounding effects affecting the placental transcriptome. Our subjects were mostly of a Caucasian background, with predominantly male sex placentas from singleton pregnancies considered in each group. All of the participants delivered spontaneously via the vaginal route, except one who delivered via a caesarean section. There were no significant differences in maternal ages, maternal BMI, gestational ages, placental weights, baby birth weights, and exposure durations between the warm and cold groups (Table 1, Figure 1C). None of the pregnant women had diabetes mellitus, hypertension, genitourinary infection, or preeclampsia at labour [19].

### 3.2. The Transcriptomic Profiles Are Altered in Preterm Placentas Delivered During Summer Months Compared to Winter Months

**Figure 2:**
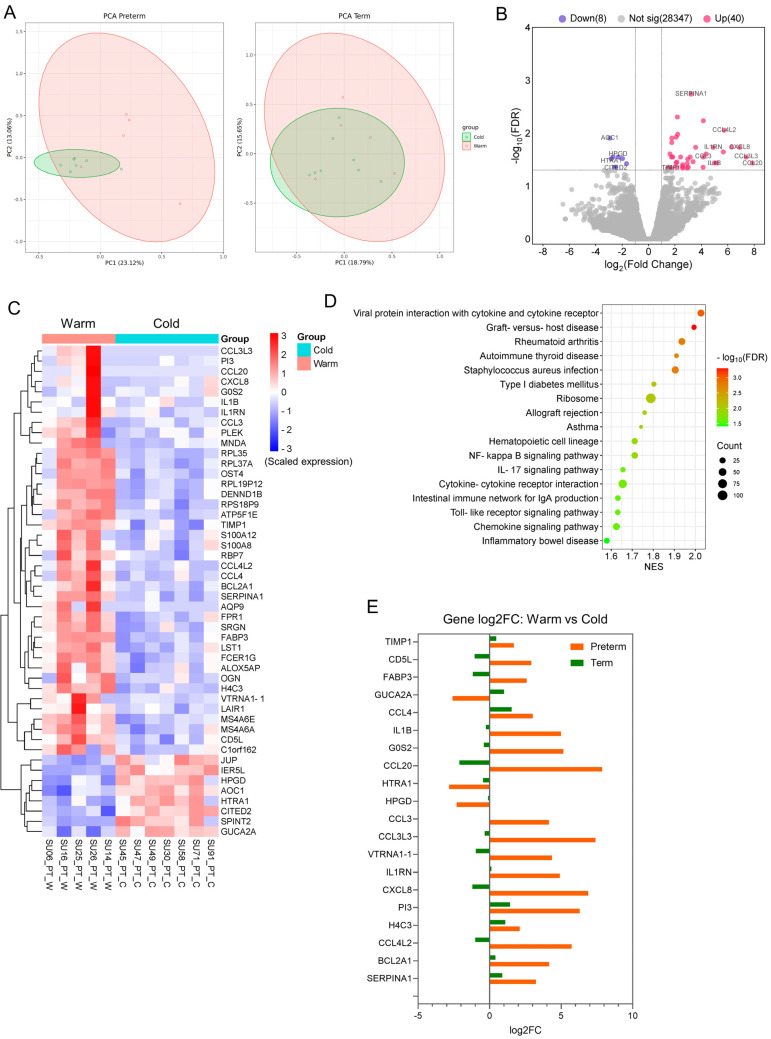
Differential gene expression analysis between warm and cold groups of placentas. (**A**) PCA plots showing group separation between warm and cold groups in preterm and term placentas. (**B**) Volcano plots showing DEGs in preterm-warm placentas compared to the preterm-cold group. (**C**) A heat map showing the expression of 48 significant DEGs in preterm-warm and preterm-cold placentas. (**D**) Signalling pathways in preterm-warm placentas positively enriched by GSEA. (**E**) The comparison of expression by a selected set of genes between term and preterm placentas.

To evaluate the molecular effects of summer warm temperatures on placentas, we conducted a differential gene expression (DE) analysis between the Preterm-warm and Preterm-cold, as well as Term-warm and Term-cold groups separately using edgeR. Our Principal Component Analysis (PCA) demonstrated clearer clustering between the warm and cold groups in preterm placentas compared to warm and cold groups in term placentas (Figure 2A).

The DE analysis identified a total of 48 significantly differentially expressed genes (DEGs) (log2FC > ±1, FDR < 0.05), with 40 genes upregulated and 8 genes downregulated (Figure 2B, Table 2 and Table S2). The majority of the top-hit upregulated genes were inflammatory cytokines and chemokines, including SERPINA1, IL1B, CCL3, CCL3L3, CCL4, CCL4L2, CCL20, and CXCL8 (FDR < 0.05) (Figure 2B, Table 2). In contrast, the downregulated DEGs were non-immune in nature. They included the diamine oxidase enzyme, which catabolises histamine (AOC1); the serine protease enzyme (HTRA1); and the enzyme that catabolises prostaglandins (HPGD) (Figure 2B, Table 2). Heatmap analysis showed the clustering of these upregulated and downregulated genes between the warm- and cold-exposure groups (Figure 2C). Conversely, the Term-warm and Term-cold groups did not demonstrate any significantly differentially expressed genes (Table S3).

Next, we performed GSEA analysis on the differentially expressed genes from preterm placentas (Preterm-warm vs. Preterm-cold) using the KEGG Pathway functional database [24]. The upregulated genes positively enriched 17 signalling pathways, which were mostly related to inflammation, infection, immune regulation, and graft rejection (FDR < 0.05). These include NF-kB (NES = 1.71), IL17 (NES = 1.65), Toll-like receptor (NES = 1.63), chemokine (NES = 1.62), and allograft rejection (NES = 1.76) signalling pathways (Figure 2D, Table S4), all of which have implications for various adverse pregnancy outcomes. No signalling pathway was significantly enriched with the downregulated genes. Our GSEA analysis, using differentially expressed genes from Term-warm vs. Term-cold placenta analysis, showed that only the steroid hormone biosynthesis signalling pathway was positively enriched (NES = 1.96, FDR = 0.001), but no negative enrichment of pathways was noted (Figure S1, Table S5).

We also compared 20 selected DEGs from the Preterm-warm vs. Preterm-cold analysis with those genes from the DE analysis of the Term-warm vs. Term-cold (Figure 2E). The expression patterns of the inflammation- and chemokine-associated genes in the preterm placentas were opposite to the expression patterns of those genes in the term placentas (Figure 2E). For instance, IL1B, CXCL8, CCL4L2, CCL3, CCL3L3, and CCL20 genes were upregulated in the Preterm-warm group compared to the Preterm-cold group, whereas these genes were downregulated in the Term-warm group compared to the Term-cold group (Figure 2E). This contrasting expression pattern suggests potential differential tissue responses to thermal exposure in term and preterm placentas, highlighting the need for further research to determine whether epigenetic or other host factors are involved in the variable responses observed in term placentas.

Together, our data revealed a distinct transcriptomic signature associated with upregulated inflammation, immune modulation, and chemokine signalling in preterm placentas delivered during the summertime (exposed to warm environmental temperature) compared to preterm placentas delivered during the wintertime (exposed to cold temperatures). This distinct signature was not observed in term placentas under similar thermal exposure conditions.

### 3.3. Functional Analysis Reveals the Enrichment of Inflammatory and Immune Cells and Their Functions in Preterm Placentas Delivered During Summertime

**Figure 3:**
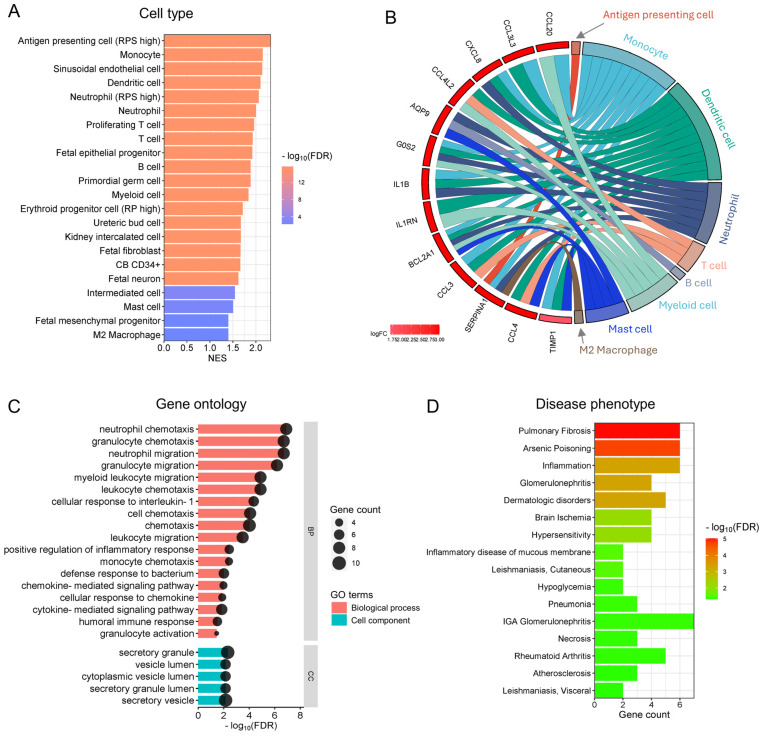
Cell-type enrichment and gene ontology analysis. (**A**) A bar plot showing the positive enrichment of cell types in preterm-warm placentas identified by GSEA. (**B**) A chord plot showing links between individual DEGs and enriched cell types. (**C**) A bar plot showing biological processes and cell components significantly enriched with the upregulated DEGs in preterm-warm placentas. (**D**) A bar plot showing disease phenotype enrichment with upregulated DEGs in the preterm-warm placentas.

Our functional cell-type enrichment analysis identified the significant positive enrichment of various inflammatory and immune cells in preterm placentas which were delivered during summer compared to those preterm placentas delivered during winter (FDR < 0.05) (Figure 3A). No negative enrichment was detected in this analysis. The positively enriched cells within the preterm placentas (chorionic villi) were predominantly antigen-presenting cells (APC)/dendritic cells, neutrophils, monocytes, T and B cells, and M2 macrophages (Figure 3A). The major genes (DEGs) associated with the enriched cell types are illustrated in the chord plot (Figure 3B). Besides immune cells, sinusoidal endothelial cells, foetal fibroblasts, and mesenchymal progenitor cells were also positively enriched in the Preterm-warm placentas (Figure 3A). No cell-type enrichment was observed in the Term-warm placentas when compared with the Term-cold group.

Our gene ontology (GO) functional analysis of the upregulated DEGs from the Preterm-warm placenta group identified several significant biological processes (FDR < 0.05), predominantly associated with neutrophil, granulocyte, monocyte, and lymphocyte chemotaxis/migration, inflammatory responses, chemokine/cytokine-mediated signalling pathways, and humoral immune responses (Figure 3C, Table S6). The major cellular components (CCs) involved in these biological processes were identified as secretory granules, secretory vesicles, vesicle lumens, and secretory granule lumens, suggesting that a secretory phenotype of the immune cells was potentially active within the preterm placentas (villous compartment) delivered during the warm seasons (Figure 3C, Table S6). Downregulated DEGs in the Preterm-warm group did not significantly identify any biological process via the GO analysis. As there were no significant DEGs detected in term placentas, GO analysis was not conducted.

Next, we performed functional enrichment analysis of disease phenotypes using the DisGeNET disease database on the DEGs of the Preterm-warm placenta group. Our analysis positively enriched several inflammatory and immune-related disease conditions (FDR < 0.05), including pulmonary fibrosis, glomerulonephritis, rheumatoid arthritis, inflammation, and hypersensitivity (Figure 3D). The data indicate that genes associated with other inflammation-related clinical conditions were also upregulated in preterm placentas delivered during the summer months, giving the results further clinical significance.

Together, our data suggest that pregnancy exposure to summertime warm temperatures may favour immune cell migration, chemotaxis, and vesicular secretion in preterm placentas, facilitating inflammatory and immune responses, which may not occur in term placentas delivered during the summer months.

### 3.4. The Genes and Signalling Pathways Upregulated in Warm-Exposed Preterm Placentas May Mechanistically Link to PTB

To investigate whether the genes and signalling pathways upregulated in preterm placentas delivered during the warmer months were also associated with the pathomechanism of preterm birth, we conducted DE and pathway enrichment analyses of preterm and term placentas delivered during warmer months (Preterm-warm vs. Term-warm). We then compared the genes (DEGs) and signalling pathways from these two groups with those derived from the Preterm-warm vs. Preterm-cold group by employing the Venn diagram approach.

**Figure 4:**
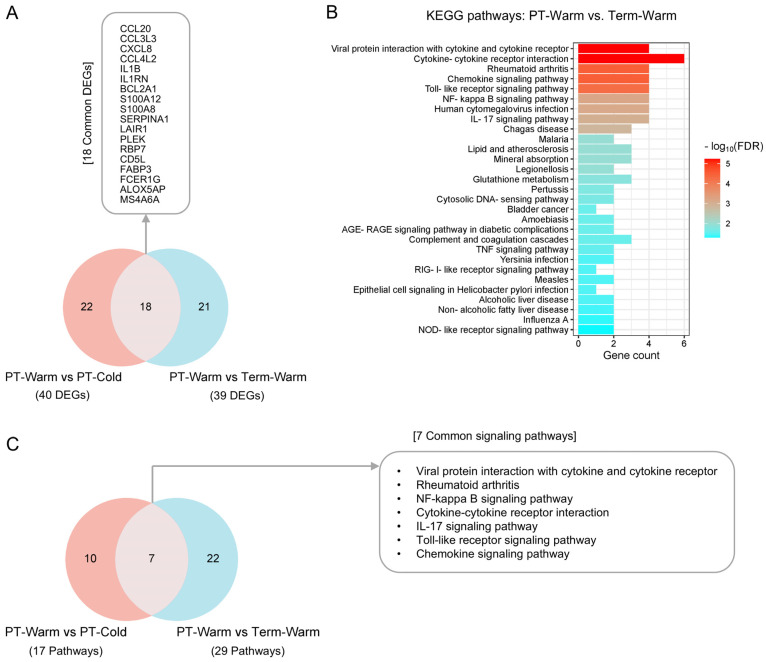
Differential gene expression and KEGG pathway analyses between preterm and term placentas. (**A**) A Venn diagram showing the overlap between significantly upregulated DEGs from the preterm-warm vs. preterm-cold and preterm-warm vs. term-warm group analyses. The common upregulated DEGs (FDR < 0.05) are given in the box. (**B**) KEGG pathway enrichment with upregulated DEGs from preterm-warm vs. term-warm placentas. (**C**) Venn diagram showing the overlap of significantly enriched KEGG signalling pathways in Preterm-warm vs. Preterm-cold and Preterm-warm vs. Term-warm group analyses. The common upregulated pathways are shown in box (FDR < 0.05).

Our DE analysis identified 54 DEGs (FDR < 0.05) in Preterm-warm placentas compared to Term-warm placentas, of which 39 were upregulated (Figure 4A, Table S7). When we compared the 40 upregulated DEGs from the Preterm-warm vs. Preterm-cold group (Table 2) with the 39 upregulated DEGs from the Preterm-warm vs. Term-warm placentas, we found 18 overlapping DEGs. Of these, 8 genes (CCL20, CCL3L3, CXCL8, CCL4L2, IL1B, IL1RN, SERPINA1 and CD5L) were primarily associated with inflammation and immune regulation (Figure 4A).

Next, we performed KEGG pathway analysis using the upregulated DEGs derived from the Preterm-warm vs. Term-warm group to explore the potential signalling pathways associated with preterm birth during warmer months. Our analysis revealed the upregulation of 29 signalling pathways in preterm placentas compared to term placentas delivered during warmer months, many of which were related to inflammation and immune responses (Figure 4B, Table S8). We then compared these 29 pathways with the 17 pathways found to be upregulated in Preterm-warm vs. Preterm-cold group analysis. This comparison identified 7 overlapping signalling pathways, all of which are primarily inflammatory in nature (Figure 4C).

Thus, our cumulative analysis identified 18 genes, and 7 signalling pathways associated with warm environmental temperature exposure, which were also upregulated in preterm placentas delivered during summer months compared to term placentas delivered during the same period (Figure 4A,C, boxes). The overlapping of these genes and signalling pathways suggests the existence of a potential mechanistic link between warm exposure and preterm labour. 

### 3.5. Key Genes Linked to Placental Inflammatory and Immune Processes Detected in Preterm Births During Summer Months

**Figure 5:**
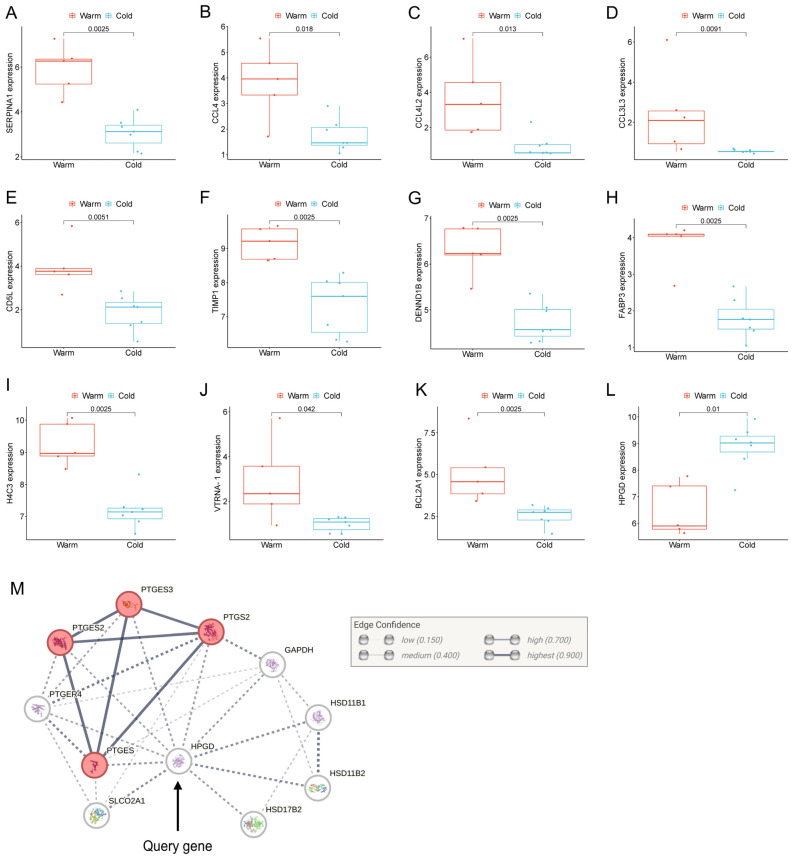
Individual gene expression in preterm placentas. (**A**–**L**) Box plots with jitters showing expressions of a selected set of DEGs in the warm and cold groups of preterm placentas. Data are presented as the median with the IQR. Each dot represents individual subjects. *p* values were determined by the Wilcoxon signed-rank test. (**M**) STRING protein–protein interaction analysis between the HPGD and related proteins (STRING v12).

Next, we selected a panel of genes based on their biological associations with inflammation, immune modulation, and placental functions, which were significantly upregulated or downregulated in the Preterm-warm placentas (Figure 5). We performed the Wilcoxon signed-rank test to compare the normalised gene expression (log2 expression values determined by edgeR) between Preterm-warm and Preterm-cold groups.

SERPINA1 was the most significantly upregulated gene in the Preterm-warm placentas compared to the Preterm-cold group, as determined by our DE analysis (FDR = 0.001, Figure 2B, Table 2). The Wilcoxon test also confirmed its significant expression (*p* = 0.002, Figure 5A). This gene was linked with the regulation of APCs, neutrophils, monocytes, and M2 macrophages, as determined by functional analysis (Figure 3A,B). SERPINA1, which encodes for α1-antitrypsin protein, is an acute-phase response protein with anti-inflammatory and immunomodulatory properties. It is predominantly produced by hepatocytes, but also by neutrophils, monocytes, and macrophages, in response to inflammation [30]

**Figure 6:**
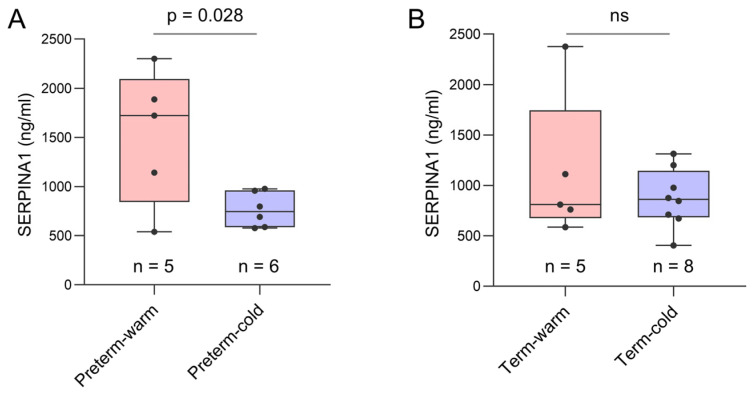
ELISA assay on placenta tissue. Box plots showing SERPINA1 protein concentrations in preterm-warm and preterm-cold placentas (**A**) and in term-warm and term-cold placentas (**B**). A protein concentration of 300 µg/mL was used as the loading concentration for each sample in the ELISA assay. Data are presented as median with interquartile range (IQR). Each dot represents an individual subject. The *p* value was determined by a two-tailed unpaired Student’s *t*-test. ns = not significant.

We conducted an ELISA assay on the same placental tissue samples used for RNA-seq analysis to confirm the expression of SERPINA1 at the protein level. The mean SERPINA1 protein concentration was ~2-fold higher in Preterm-warm placentas compared to Preterm-cold placentas (1518 ng/mL vs. 764.2 ng/mL; *p* = 0.028) (Figure 6A). The SERPINA1 protein concentration was 29% higher in Term-warm placentas than in the Term-cold placentas, although this difference was not statistically significant (Figure 6B). Cumulatively, these protein data are consistent with our RNA-seq findings.

**Figure 7:**
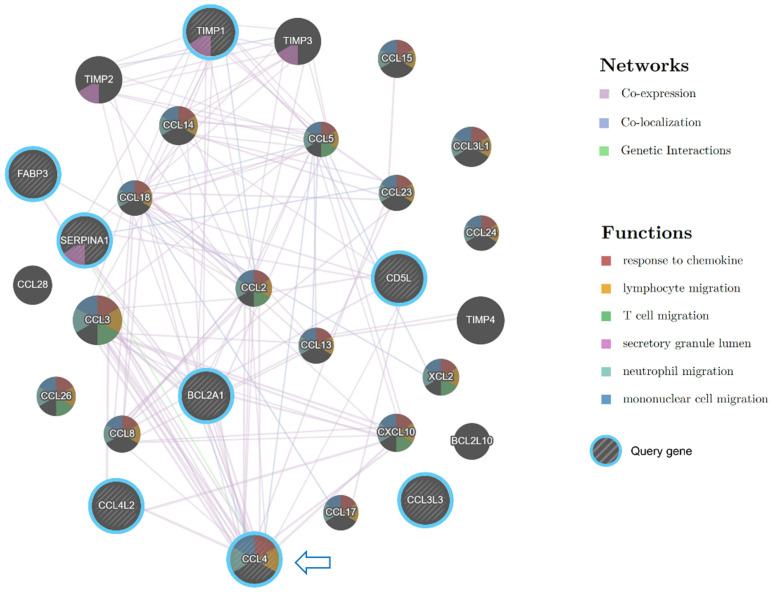
Gene interaction network analysis by Cytoscape plug-in GeneMANIA. Striped bigger nodes indicate DEGs upregulated in preterm-warm placentas. Colour codes inside each node indicate their biological function as stated in the Functions legend. Coloured edges and their connections with other genes indicate the nature of interactions.

Among the upregulated chemokines in the Preterm-warm placentas, CCL4 was the most highly upregulated (log2FC = 3; Figure 5B). CCL4 is a potent chemokine released by monocytes, lymphocytes, neutrophils, fibroblasts, and endothelial cells. It regulates immune and inflammatory responses by attracting a diverse population of immune cells [31,32,33]. The gene network analysis identified CCL4 as one of the genes most functionally connected with other immune regulatory genes (Figure 7).

CD5L, which encodes a soluble glycoprotein of the SRCR superfamily and modulates macrophage activities in inflammatory processes via macrophage polarisation towards the M2 phenotype [34,35], was significantly upregulated in the Preterm-warm placentas, where M2 macrophage enrichment was also noted (FDR = 0.005) (Figure 2, Figure 3A and Figure 5E, Table 2). Another emerging immunomodulator, TIMP1, which fosters the inflammatory process via its cytokine activity, was significantly overexpressed in the Preterm-warm placentas (Figure 5F) [36]. DENND1B, a T cell receptor (TCR) internalisation regulator in TH2 cells, was also upregulated in Preterm-warm placentas (Figure 5G, Table 2).

Fatty acid-binding proteins (FABPs) are involved in fatty acid metabolism and trafficking in the placenta. FABP3 and FABP6 proteins have been found to be increased within the placental barrier in obese pregnant mice, enhancing fatty acid transport to the foetus [37]. In our analysis, FABP3 was significantly upregulated in Preterm-warm placentas compared to the Preterm-cold group (Figure 5H, Table 2). In the Term-warm placenta group, this gene was downregulated (though not significantly) (Figure 2E). 

One of the four core histone genes, H4C3, encodes a protein that forms an octamer and regulates chromatin organisation. It was upregulated in the preterm placentas exposed to warm temperatures (Figure 5I, Table 2). Among other non-immune-associated genes, VTRNA1-1 and BCL2A1, which are associated with impaired autophagy and apoptosis, respectively, were significantly upregulated in the Preterm-warm placentas compared to the Preterm-cold group, (Figure 5J,K).

15-hydroxyprostaglandin dehydrogenase (HPGD) catalyses prostaglandins, resulting in their functional inactivation [38]. In our DE analysis and Wilcoxon test, HPGD was significantly downregulated in the Preterm-warm placentas compared to Preterm-cold placentas (Figure 2C and Figure 5L, Table 2). Due to its diverse effects on prostaglandin E synthases (STRING analysis, Figure 5M), the inactivation of this gene may augment prostaglandin-driven mechanisms, including inflammation and the initiation of labour [39].

## 4. Discussion

As pregnancy progresses, internal heat production rises due to foetal and placental metabolism; however, the dissipation of this excess heat is limited by increased maternal body mass and a relative reduction in body surface area [40]. A growing body of evidence strongly links maternal exposure to heat, particularly during the second and third trimesters, with increased risks of spontaneous preterm birth across diverse geographical regions, including the West, Sub-Saharan Africa, and Asia [3,4,5,6,7,8,9,10]. Animal model studies have postulated that high ambient temperatures alter maternal systemic thermoregulation capacity, resulting in cortisol release and subsequent oxytocin-induced uterine contractions and labour [5,8,41,42]. However, the effects of maternal heat exposure on placental molecular functions are not yet fully understood.

Here, we provide the first human report of the significant alteration of the placental transcriptome in cases of idiopathic spontaneous preterm births occurring during summer compared to the winter months. These transcriptomic alterations were primarily associated with placental inflammation, immune regulation, and allograft rejection signalling pathways, driven by inflammatory and immune cells, including neutrophils, monocytes, M2 macrophages, T and B lymphocytes, and dendritic cells, likely originating from the villous placenta. Additionally, the genes and signalling pathways upregulated in preterm placentas exposed to summertime warm temperatures overlapped with those upregulated in preterm-warm placentas compared to term-warm placentas. These shared genes and pathways are predominantly inflammatory in nature and may be involved in preterm birth mechanisms, particularly those occurring during the summer months.

This observation was noted in preterm placentas from pregnant women who delivered during the British summer period, with a mean temperature T_mean_ = 14.92 °C and an average maximum temperature T_max_ = 20.47 °C. For the comparable cold group, the mean temperature was 5.14 °C, with a maximum temperature of 8.37 °C. It was estimated that the women experienced these temperatures for an average of 9 weeks during the warmer months and 7.3 weeks during the colder months preceding parturition. Although these higher temperatures might be perceived as within a comfortable range, in colder geographical regions such as South Yorkshire, where people are naturally acclimatised to colder temperatures, a rise of 10–12 °C during the summertime could cause discomfort or heat exhaustion for pregnant women, as previously reported [5,8,43]. Accounting for its central role in maintaining foetal growth and gestation, this thesis formed the basis of our studies seeking to evaluate the effects of environmental temperature on placental function and preterm birth. These effects were driven by the cellular elements within the villous compartment, including cytotrophoblasts, syncytiotrophoblasts, extravillous trophoblasts, mesenchymal cells, endothelial cells, and immune cells [14]. 

The RNA-seq datasets used in this study were generated from the villous tissue (devoid of decidua and maternal blood) of the placentas containing the aforementioned cell populations, as demonstrated by our previous work [27]. Our functional enrichment analysis on the basis of ‘cell-type’ categories did not show enrichment for trophoblast cells but did show positive enrichment for inflammatory and immune cells, as well as endothelial, mesenchymal, and foetal epithelial progenitor cells in the preterm-warm placentas. This differential enrichment suggests an immunomodulatory response of the preterm placenta to exposure to warm temperatures during pregnancy. This inference is supported by our gene ontology analysis, showing the enrichment of cellular components associated with vesicular secretion, but not locomotion. Therefore, we speculate that the placental resident immune and parenchymal cells released chemokines and inflammatory cytokines, which attract maternal immune cells into the placenta and disrupt the normal immune balance.

The placenta samples, used in this study, were collected from pregnant women who showed no signs of genitourinary tract infection or preeclampsia at the time of delivery. Furthermore, our unpublished in-house shotgun metagenomic sequencing analysis did not detect any pathogenic bacterial or viral infections in the preterm-warm placentas used in this study. Thus, it is unlikely that the upregulation of inflammatory genes and signalling pathways in preterm placentas delivered during the summer months was due to pre-existing placental inflammation or infection. However, due to the etiological and mechanistic complexities, as well as the intricate taxonomic classification of spontaneous PTB, we were unable to determine whether other factors, such as sterile placental infection, foetal anomalies, or uterine distension, contributed to the findings presented in the warm-exposed preterm placentas [44]. We noted a relatively higher degree of sample heterogeneity in preterm placentas delivered during the summertime compared to those delivered in the winter. No specific phenotype of PTB was associated with the timing of delivery.

While there is a paucity of research on the molecular effects of heat exposure on the human placenta, one study has shown that maternal stress due to climate-related disasters such as hurricanes altered the placental transcriptome and was linked to infant temperament. This altered transcriptome also demonstrated an enrichment of functional pathways related to inflammation, extracellular matrix integrity, and sensory perception [45]. A mouse model study demonstrated that maternal exposure to cold ambient temperatures (18 °C vs. 28 °C) was associated with the upregulation of genes involved in antigen processing and presentation, cytokine receptor interaction, and complement activation, and the downregulation of genes involved in oxidative phosphorylation, myofibril assembly, and muscle contraction [46]. These findings have similarities with what we observed in our Preterm-warm placentas (Figure 2 and Figure 3). However, it is important to note that we did not detect any significant transcriptomic alterations in the term placentas exposed to similar thermal conditions. The reasons for this difference are not apparent and warrant further study.

Our cross-comparative analysis of genes and signalling pathways between term and preterm placentas (delivered during summer) and between preterm-warm and preterm-cold groups identified 18 overlapping upregulated genes and 7 signalling pathways, including IL-17, NF-κB, Toll-like receptors, and chemokine signalling pathways.

The placental NF-κB signalling pathway remains downregulated to maintain pregnancy. Its activation initiates a cascade of events that facilitate the onset of labour through the production of pro-inflammatory cytokines, chemokines, and prostaglandin-synthesising enzymes [47]. Infiltrating immune cells in the villous placenta secrete chemokines and cytokines, leading to the activation of NF-κB [48]. The premature aberrant activation of NF-κB signalling has been implicated in PTB [49]. This putative activation of NF-κB signalling by the infiltrating immune cells aligns with our observation that heat-exposed preterm placentas may favour the chemotaxis of immune cells from maternal circulation to those from the placenta.

Toll-like receptor (TLR) signalling and its functional activation have been shown to increase in PTB [50]. The stimulation of TLRs induces the release of pro-inflammatory cytokines in the foetal membrane and placenta [51,52]. The activation of TLR signalling leads to uterine contractions, cervical ripening, and preterm premature rupture of membranes (PPROM) in PTB [50]. Therefore, it is likely that heat exposure during pregnancy may amplify the risk of preterm labour via the upregulation of TLR signalling, as we have demonstrated. Additionally, studies have shown that IL17, produced by T cells, promotes inflammation at the fetomaternal interface of the placenta in PTB [53]. Our analyses showed that a warm environment positively enriched both T cells and IL-17 signalling in the preterm placenta. Therefore, this ‘T cell-IL17 signalling axis’ could be linked to the detrimental effects on placental function associated with thermal exposure.

Out of the 48 differentially expressed genes, we identified 12 genes as being important mediators in thermal responses based on their expression patterns and relevance to pregnancy and placental functions (Figure 5). Among them, SERPINA1, which encodes for α1-antitrypsin, stands out as a potential biomarker target. Increased levels of α1-antitrypsin were detected in the villous placenta of preeclampsia patients, where it was associated with syncytiotrophoblast destruction and secretion into urine [54]. The detection of placenta-originating α1-antitrypsin in urine was suggested as a potential biomarker for the assessment of the severity of preeclampsia [54]. Interestingly, α1-antitrypsin expression at the gene and protein levels was decreased in cases of spontaneous PTB compared to cases of term birth [55]. Conversely, a recent study demonstrated a significant increase in α1-antitrypsin levels in maternal blood from spontaneous PTB compared to term birth during the 1st and 2nd trimesters, though no difference was observed at delivery [56].

In our analysis, SERPINA1 was the most significantly upregulated gene in the preterm-warm group of placentas compared to the corresponding cold group, which was also true for its protein level expression (Figure 6), suggesting its potential as a marker of the placental response to high-heat exposure in high-risk pregnancies.

Heat stress and heat acclimation can induce epigenetic modifications such as DNA methylation and histone modifications, which help to protect cells from thermal damage by adjusting the transcriptional levels of heat-responsive genes [57]. In our study, although no classical heat-responsive genes such as heat-shock proteins were differentially expressed, we observed the significant upregulation of the core histone gene H4C3 in preterm-warm placentas. The H4C3 protein plays a critical role in nucleosome formation and chromatin organisation, regulating DNA replication and transcription. Variants of this gene have been associated with various pathologies, including neurodevelopmental disorders [58]. The thermal responsiveness of H4C3 in the context of placental pathology remains unclear and warrants further investigation.

Prostaglandins play pivotal roles in initiating human parturition by promoting uterine contraction and cervical ripening, and their inhibition can delay labour [39,59]. The enzyme HPGD catalyses the functional inactivation of prostaglandins [38]. In our study, HPGD was significantly downregulated in preterm placentas exposed to warm environmental temperatures. Placental HPGD has been reported to be markedly downregulated in both animal models and cases of human preterm birth [60,61]. It is therefore plausible that the suppression of HPGD induced by warm temperatures may predispose patients to premature labour, although this hypothesis requires validation through further studies.

### Study Limitations

Although our findings align with transcriptomic data from controlled thermal exposure animal studies, our conclusions are based on a relatively small sample size with relatively low statistical power. In addition, due to the limited sample size and lack of metadata, we were unable to perform multivariate analysis incorporating potential confounding factors such as dietary quality, sun exposure, physical activity, and exposure to environmental toxins, all of which may influence placental transcriptomic profiles. Temperature exposure for our study participants was estimated using regional weather data. Including direct measurements of the body and domiciliary temperatures of the pregnant women during their third trimester, in addition to regional weather data, could provide a more accurate assessment of temperature exposure. However, there is increasing evidence indicating that pregnant women are more likely to experience heat exhaustion during summer compared to winter across various geographical regions [5,8,43], which is consistent with our observations.

## 5. Conclusions

Maternal exposure to elevated summer environmental temperatures during the third trimester of pregnancy was associated with altered placental transcriptomes, and was seen predominantly in cases of spontaneous preterm birth, but not in term births. This transcriptomic alteration was associated with placental inflammation, immune regulation, and allograft rejection. However, it remains uncertain whether these placental responses were a direct effect of elevated temperatures on the placenta or secondary to maternal systemic responses to warm exposure. Our data revealed a potential detrimental impact on placental functions due to long-term exposure to moderately elevated temperatures during pregnancy. These findings warrant further large-scale molecular studies across diverse geographical regions to clarify the effects of heat on placental function. This research is crucial for combating the impacts of global climate change on pregnancy outcomes.

## Figures and Tables

**Table 1:** Study participants’ criteria whose RNA-seq data were analysed (*n* = 25). Data are presented as mean ± standard deviation. *p* values were determined by either the Mann–Whitney U test or ^#^ Fisher’s Exact Test. ns = not significant.

	Term (*n* = 13)		*p* Values	Preterm (*n* = 12)		*p* Values
	Warm (*n* = 5)	Cold (*n* = 8)		Warm (*n* = 5)	Cold (*n* = 7)	
Monthly mean weather temp at delivery (°C)	16.06 ± 1.12	5.18 ± 0.53	0.0008	14.92 ± 1.63	5.14 ± 0.61	0.0025
Monthly max weather temp at delivery (°C)	20.69 ± 0.90	8.2 ± 1.31	0.0016	20.47 ± 0.73	8.37 ± 1.40	0.0013
Mean exposure duration prior delivery (days)	51.6 ± 25.4	44.63 ± 39.77	0.8057	64.2 ± 31.15	51.14 ± 38.64	0.5025
Gestational age at delivery (weeks)	39.9 ± 1.1	39.7 ± 1.0	ns	30.9 ± 4.1	33.3 ± 3.6	ns
Maternal age (years)	32.4 ± 5.5	32.0 ± 4.6	ns	29.9 ± 8.3	26.2 ± 5.2	ns
BMI at delivery (kg/m^2^)	25.6 ± 2.9	27.2 ± 4.8	ns	32.2 ± 18.2	26.7 ± 4.9	ns
Placental weight (g)	603.9 ± 141.3	729 ± 122.5	ns	512 ± 167.9	447.3 ± 102.5	ns
Baby birth weight (kg)	3.3 ± 0.4	3.6 ± 0.2	ns	1.7 ± 0.7	2.0 ± 0.7	ns
*Foetal sex, n (%)*						
Male	3 (60%)	6 (75%)	ns ^#^	4 (80%)	4 (57%)	ns ^#^
Female	2 (40%)	2 (25%)		1 (20%)	3 (43%)	
*Maternal ethnicity, n (%)*						
White Caucasian	5 (100%)	6 (75%)	-	4 (80%)	7 (100%)	-
Black	0 (0%)	2 (25%)		0 (0%)	0 (0%)	
Asian and others	0 (0%)	0 (0%)		1 (20%)	0 (0%)	

**Table 2:** Table showing 48 significantly differentially expressed genes (DEGs) (FDR < 0.05) in preterm placentas exposed to warm environmental temperature, of which 40 DEGs were upregulated and 8 DEGs were downregulated. Genes are ranked based on log2FC in descending order.

Gene Symbol	Gene Name	log2FC	FDR
Upregulated DEGs			
CCL20	C-C motif chemokine ligand 20	7.868273398	0.036752803
CCL3L3	C-C motif chemokine ligand 3 like 3	7.395239339	0.028298209
CXCL8	C-X-C motif chemokine ligand 8	6.90348151	0.018563659
PI3	peptidase inhibitor 3	6.299703044	0.018354829
CCL4L2	C-C motif chemokine ligand 4 like 2	5.735427056	0.00883849
AQP9	aquaporin 9	5.664779974	0.02266962
G0S2	G0/G1 switch 2	5.165543412	0.036752803
IL1B	interleukin 1 beta	4.988398402	0.036752803
IL1RN	interleukin 1 receptor antagonist	4.898668294	0.018563659
VTRNA1-1	vault RNA 1-1	4.361248506	0.024808644
BCL2A1	BCL2-related protein A1	4.153647206	0.005843209
CCL3	C-C motif chemokine ligand 3	4.150877295	0.028298209
OGN	osteoglycin	4.133529937	0.044324798
S100A12	S100 calcium-binding protein A12	3.570600928	0.018563659
S100A8	S100 calcium-binding protein A8	3.371015213	0.034733036
SERPINA1	serpin family A member 1	3.229324877	0.001820221
LAIR1	leukocyte-associated immunoglobulin like receptor 1	3.150781231	0.028298209
CCL4	C-C motif chemokine ligand 4	3.013097884	0.044324798
PLEK	pleckstrin	2.964301423	0.031052031
RBP7	retinol-binding protein 7	2.927368252	0.0378486
CD5L	CD5 molecule like	2.906771556	0.04583665
FABP3	fatty acid-binding protein 3	2.585251055	0.045703663
MS4A6E	membrane spanning 4-domains A6E	2.584792006	0.039727763
C1orf162	chromosome 1 open reading frame 162	2.576776932	0.043170704
MNDA	myeloid cell nuclear differentiation antigen	2.431885444	0.028298209
FCER1G	Fc epsilon receptor Ig	2.206550903	0.010524081
RPL35	ribosomal protein L35	2.184858984	0.004952226
LST1	leukocyte specific transcript 1	2.145185426	0.035237296
ALOX5AP	arachidonate 5-lipoxygenase activating protein	2.103598893	0.044324798
H4C3	H4 clustered histone 3	2.094398124	0.011879223
FPR1	formyl peptide receptor 1	2.082935671	0.036752803
MS4A6A	membrane spanning 4-domains A6A	1.832523756	0.028298209
RPS18P9	ribosomal protein S18 pseudogene 9	1.768847282	0.012395102
RPL19P12	ribosomal protein L19 pseudogene 12	1.760969738	0.015050412
RPL37A	ribosomal protein L37a	1.75489983	0.028298209
TIMP1	TIMP metallopeptidase inhibitor 1	1.700953541	0.04583665
DENND1B	DENN domain containing 1B	1.627129554	0.024808644
SRGN	serglycin	1.573143772	0.044307338
ATP5F1E	ATP synthase F1 subunit epsilon	1.443927266	0.04583665
OST4	oligosaccharyltransferase complex subunit 4, non-catalytic	1.434266824	0.049762171
Downregulated DEGs			
AOC1	amine oxidase copper containing 1	−2.934503787	0.012395102
HTRA1	HtrA serine peptidase 1	−2.826833202	0.030657503
IER5L	immediate early response 5 like	−2.694111567	0.028298209
GUCA2A	guanylate cyclase activator 2A	−2.577359202	0.044324798
CITED2	Cbp/p300 interacting transactivator with Glu/Asp rich carboxy-terminal domain 2	−2.48474137	0.044324798
HPGD	15-hydroxyprostaglandin dehydrogenase	−2.301054733	0.028298209
JUP	junction plakoglobin	−1.970586571	0.030191088
SPINT2	serine peptidase inhibitor, Kunitz type 2	−1.666023242	0.0378486

## Data Availability

Additional data can be found in the Supplemental Files. The previously published RNA-seq datasets used in this manuscript can be found in the NCBI Gene Expression Omnibus (GEO) repository using accession number GSE211927 (Supplementary Table S1). Any additional data from this study are available from the corresponding author upon reasonable request.

## Supplementary Materials

The following supporting information can be downloaded at https://www.mdpi.com/article/10.3390/cells14040303/s1. Figure S1: GSEA on the differentially expressed genes from the DE analysis between Term-warm (*n* = 5) vs. Term-cold (*n* = 8) placenta samples. Table S1: KEGG pathways enrichment: Preterm-warm vs. Term-warm; Table S2: edgeR DE analysis: Preterm-warm vs. Preterm-cold; Table S3: edgeR DE analysis: Term-warm vs. Term-cold; Table S4: GSEA with Preterm-warm vs. Preterm-cold DEGs; Table S5: GSEA with Term-warm vs. Term-cold DEGs; Table S6: GO: Biological processes; Table S7: edgeR DE analysis: Preterm-warm vs. Term-warm; Table S8: KEGG pathways enrichment: Preterm-warm vs. Term-warm.
