# Supplementary material for: Seasonal Influences on Human Placental Transcriptomes Associated with Spontaneous Preterm Birth
Source: Cells. 2025 Feb 18;14(4):303. doi: 10.3390/cells14040303 (PMC11853885; doi:10.3390/cells14040303)
Supplement: Supplementary file 1 [file cells-14-00303-s001.zip › Akram et al Supplemental file.pdf]

Supplemental figure

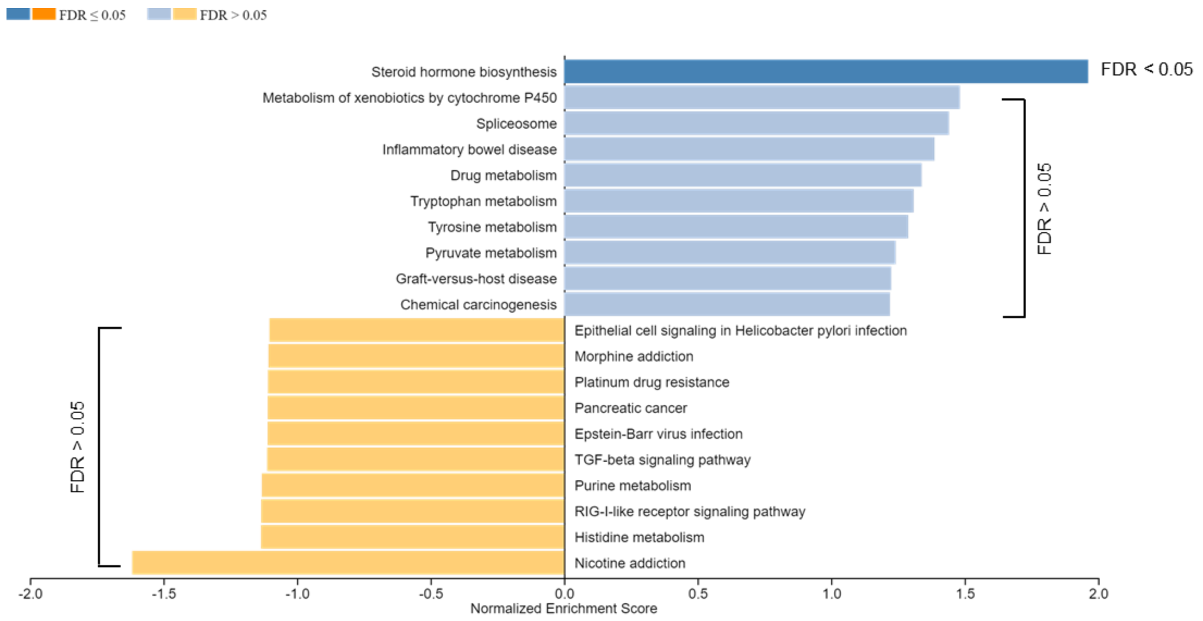

Figure S1: GSEA on the differentially expressed genes from the DE analysis between Term-warm (n = 5) vs. Term-cold (n = 8) placenta samples.
